# Factors Associated with Increased Knowledge about Breast Density in South Australian Women Undergoing Breast Cancer Screening

**DOI:** 10.3390/cancers16050893

**Published:** 2024-02-23

**Authors:** Avisak Bhattacharjee, David Walsh, Pallave Dasari, Leigh J. Hodson, Suzanne Edwards, Sarah J. White, Deborah Turnbull, Wendy V. Ingman

**Affiliations:** 1 Discipline of Surgical Specialties, Adelaide Medical School, The Queen Elizabeth Hospital, University of Adelaide, Woodville South, SA 5011, Australia; avisak.bhattacharjee@adelaide.edu.au (A.B.); david.walsh2@sa.gov.au (D.W.); pallave.dasari@adelaide.edu.au (P.D.); leigh.hodson@adelaide.edu.au (L.J.H.); 2 Robinson Research Institute, University of Adelaide, Adelaide, SA 5006, Australia; 3 School of Public Health, University of Adelaide, Adelaide, SA 5005, Australia; suzanne.edwards@adelaide.edu.au; 4 Centre for Social Impact, University of New South Wales, Sydney, NSW 2052, Australia; sarah.white@unsw.edu.au; 5 School of Psychology, University of Adelaide, Adelaide, SA 5005, Australia; deborah.turnbull@adelaide.edu.au

**Keywords:** breast density, mammographic density, knowledge, mammography, breast cancer screening, breast cancer risk

## Abstract

**Simple Summary:**

Breast density is an independent risk factor for breast cancer and can impede detection of cancer by mammography. There is growing awareness of breast density in Australia and globally, but it is unclear whether this awareness is increasing knowledge of what breast density is and what it means to have dense breasts. This study was conducted to investigate South Australian women’s knowledge of the common facts and misconceptions about breast density. This study reports that women who had previously heard the term breast density had increased knowledge compared to those who had not, suggesting that current efforts to raise awareness are leading to better knowledge. Despite this, the study shows that there are widespread misconceptions that must be actively dispelled, including the misunderstanding that breast density can be determined by touch.

**Abstract:**

**Background:** There is growing awareness of breast density in women attending breast cancer screening; however, it is unclear whether this awareness is associated with increased knowledge. This study aims to evaluate breast density knowledge among Australian women attending breast cancer screening. **Method:** This cross-sectional study was conducted on women undergoing breast cancer screening at The Queen Elizabeth Hospital Breast/Endocrine outpatient department. Participants were provided with a questionnaire to assess knowledge, awareness, and desire to know their own breast density. **Result:** Of the 350 women who participated, 61% were familiar with ‘breast density’ and 57% had ‘some knowledge’. Prior breast density notification (OR = 4.99, 95% CI = 2.76, 9.03; *p* = 0.004), awareness (OR = 4.05, 95% CI = 2.57, 6.39; *p* = 0.004), younger age (OR = 0.97, 95% CI = 0.96, 0.99; *p* = 0.02), and English as the language spoken at home (OR = 3.29, 95% CI = 1.23, 8.77; *p* = 0.02) were independent predictors of ‘some knowledge’ of breast density. A significant proportion of participants (82%) expressed desire to ascertain their individual breast density. **Conclusions:** While knowledge of breast density in this Australian cohort is generally quite low, we have identified factors associated with increased knowledge. Further research is required to determine optimal interventions to increase breast density knowledge.

## 1. Introduction

Breast density, also known as mammographic density, refers to the radiological appearance of the breast on an X-ray mammogram. High breast density appears on a mammogram as white areas and signifies a high abundance of fibroglandular tissue in the breast in proportion to the abundance of adipose tissue. Breast density is typically quantified as ‘percent mammographic density’, calculated as the ratio of the area of dense breast tissue to the total area of breast tissue [1,2]. Breast density impacts upon both breast cancer risk and the early detection of breast cancer on a mammogram and has become a major focus of interest within the medical, scientific, and broader community [3,4]. Given the clinical implications, there is ongoing consideration of how best to incorporate breast density into screening recommendations for organised breast screening programs [5].

The American College of Radiology defines four categories of breast density in the 5th edition of Breast Imaging-Reporting Data System (BI-RADS): Category A (Mostly fatty), Category B (Scattered density), Category C (Heterogeneously dense), and Category D (Extremely dense) [6]. Within this classification, the ‘Heterogeneously dense’ and ‘Extremely dense’ categories are sometimes grouped together as ‘dense breasts’, although the studies that have informed current recommendations about risk and screening focus on the extremely dense category and are not generalizable to those with heterogeneously dense breast tissue [7]. Approximately 8% of women within the age range of 40 to 74 have breast tissue categorised as extremely dense and 35% have heterogeneously dense tissue [8]. The BI-RADS classification system is subjective and leads to inconsistencies in measurement [9], which is why new methods for quantitative assessment of breast density are now available, including Volpara and Quantra software [10].

The sensitivity of mammography to detect cancer among individuals with dense breast tissue is reduced compared to those with low density. A recent study, employing the Incremental Cancer Detection Rates (ICDR), estimated that approximately 267,000 mammographically occult undetected tumours may be present among women with dense breast tissue who participated in a population-based screening program in 2021 in the United States [11]. A tumour missed in a dense breast could result in unfavourable consequences such as an interval cancer, which is a breast cancer diagnosis after a negative screening mammogram and before the next mammogram is due [12]. Conceivably, this cancer might have been pre-existing during the unremarkable mammogram, concealed beneath the more radiopaque and whiter background of high breast density. Up to fifty percent of cancers detected through mammography may evade detection due to the masking influence exerted by extremely dense breast tissue [13].

In addition to causing reduced sensitivity of mammography to detect cancer, breast density is also an independent risk factor for breast cancer. Women categorised with extremely dense breasts have a 4–6-fold greater risk of breast cancer compared to those categorised with mostly fatty breasts when matched with age and BMI [14,15,16]. Up to 35% of pre-menopausal breast cancer and 16% of post-menopausal breast cancers can be attributed to breast density [17]. Moreover, while not conclusively established [18], breast density may also increase breast cancer-specific mortality [19]. 

Considering the significance of breast density in risk and detection of breast cancer, the Food and Drug Administration, in a statement dated 9 March 2023, announced the revision of its mammography regulations, necessitating the incorporation of breast density information into facility reports [20]. The European Union Society of Breast Imaging recently released screening recommendations for women with extremely dense breasts, stating that “women should be appropriately informed about their individual breast density... and on the diagnostic and prognostic implications of having dense breasts” [21]. 

In Australia, breast cancer screening is conducted largely within the BreastScreen Australia public screening program. The policies of both BreastScreen Australia and the Royal Australian and New Zealand College of Radiologists are to not report breast density within the population-based screening program [3,22,23]. However, BreastScreen Western Australia, BreastScreen South Australia, and the majority of private screening providers currently incorporate breast density notification into their screening protocol. For over a decade, BreastScreen Western Australia has notified women who have dense breasts. The notification includes an explanation about how high density can potentially impede cancer detection and provides participants with a website link with further explanation of breast density, encompassing its potential associated risks [3,24]. More recently, BreastScreen South Australia commenced notification of breast density to all screening participants commencing August 2023, following the successful execution of a pilot study [25].

However, breast density notification may not achieve the intended positive outcomes if there exists a lack of understanding about what breast density is. Furthermore, there may be common misconceptions about breast density that impede people’s knowledge. Currently, there is limited research on the knowledge of Australian women about breast density. One study evaluated knowledge in women attending BreastScreen Western Australia, finding that, among the cohort of breast density notified women, 85% knew about the reduced sensitivity of detecting cancer on a mammogram due to breast density, a significant contrast to the non-notified group, where 54% knew about this reduced sensitivity [24]. Furthermore, 25% of notified women were cognizant of the increased breast cancer risk associated with dense breasts, which stands in contrast to the 13.2% knowledge rate observed in the non-notified group. In the present study, we aimed to assess awareness and knowledge about breast density in a South Australian cohort, and investigate whether women want to know their breast density. This study was conducted prior to the recent change in policy by BreastScreen South Australia and may serve as a baseline for future investigations assessing the impact of widespread notification on breast density knowledge.

## 2. Materials and Methods

This cross-sectional study was conducted in women attending the outpatient department of the Breast/Endocrine Surgical unit of The Queen Elizabeth Hospital between March 2022 and July 2023 (CALHN Ethics approval reference number 15681). The study was completed just prior to the introduction of breast density notification policy by BreastScreen South Australia, which was implemented in August 2023 [25]. The Queen Elizabeth Hospital is the second-largest tertiary healthcare institution in South Australia, addressing the medical needs of around 244,000 individuals spanning seventeen suburbs in the western region of the city. Of this population, 51% are female. The median weekly household income among residents in private accommodations is 1516 AUD and 68% of the inhabitants within the hospital’s catchment area predominantly speak in English for household communication [26].

### 2.1. Survey Procedure

All women who attended the outpatient department for breast screening during the study period were invited to participate. Participants were provided with a questionnaire about breast density awareness and knowledge. The questionnaire was based on a questionnaire designed to assess breast density knowledge in a Western Australian cohort [24]. All the women in the waiting bay who were attending the clinic for a screening mammogram were actively engaged. Women who appeared to be distressed to the researcher (AB) and those lacking the capability to provide informed consent were ineligible to participate. 

Sociodemographic and clinical data from the patient database were retrieved from the Electronic Medical Record. These data included age, suburb of residence (extracted to determine Accessibility/Remoteness Index of Australia-ARIA and Socio-Economic Index for Areas-SEIFA), screening frequency, and breast density status measured by Volpara software version 3.4. Language spoken at home was self-reported through the questionnaire. 

The questionnaire assessed recognition of the term ‘breast density’, prior screening and breast density notification, whether participants wanted to know their breast density, as well as five key questions that assessed knowledge about breast density (see the Supplementary File). Participants who answered one key knowledge question correctly or less were defined as having ‘low knowledge’; those who answered two and above correctly were defined as having ‘some knowledge’ about breast density. The categories of ‘low knowledge’ and ‘some knowledge’ were established by the research team following a discussion, where a consensus was reached that this approach would provide a sensible and pragmatic representation of women’s knowledge.

### 2.2. Statistical Analysis

The participants’ sociodemographic characteristics including age, language spoken at home, Socio-Economic Index of Australia (SEIFA), Accessibility/Remoteness Index of Australia (ARIA), and clinical profile including number of mammograms in the last 3 years and breast density status within the cohort were expressed in frequency and percentages. The data regarding the women who had ever heard the term ‘breast density’ and ‘who were told about their own breast density by any health professionals’ were also calculated for descriptive representation. 

Both sociodemographic and clinical variables were tested as predictors of ‘some knowledge’ about breast density by multivariable binary logistic regression analysis. Response of the participants to each key question and their interest to know about own density was individually tested against the significant predictors of knowledge by multivariable binary logistic regression analysis. 

Missing data were managed using an imputation method. Specifically, in instances where categorical variables exhibited missing values, imputation was performed by substituting these values with the mode of the respective category. Categorical variables were presented using frequency and percentage, while continuous variables were summarised using mean and standard deviation. To evaluate the relationship between dependent and independent variables, odds ratios (OR) were employed. The precision of these estimates was further evaluated through the calculation of 95% confidence intervals (95% CI). Significance testing was conducted using Fisher’s exact test and the chi-square test to determine the *p*-value (<0.05 being classed as statistically significant). All the determined *p*-values were adjusted using Benjamini–Hochberg method. Analyses were performed using IBM SPSS statistics version 28. 

## 3. Results

Overall, 427 women were invited to participate, with 346 women providing immediate responses. Six women opted to take the questionnaire home to complete at their convenience and subsequently mail it back; however, only four of them returned the questionnaire. In sum, 350 ultimately responded to the questionnaire, yielding a response rate of 82%.

Table 1 provides a comprehensive summary of the baseline characteristics encompassing sociodemographic variables and knowledge status, prior awareness of the term ‘breast density’, and desire to know their breast density. The mean age of the respondents was 61 years (SD 11.45). Seventy percent of respondents were in the current Australian breast screening target age group of 50–74 years, with 17% aged between 40–49 years, and 13% aged 75 and older. Most respondents reported English as their primary language (94%). A substantial 97% of the cohort resided in major cities, according to the Accessibility/Remoteness Index of Australia (ARIA), signifying they were located in highly accessible, accessible, or moderately accessible areas (Table 1).

Table 2 provides a synopsis of the factors associated with ‘some knowledge’ about breast density, defined as a score of two or more out of five questions answered correctly. Younger age, English as the language spoken at home, prior awareness of the term ‘breast density’, and prior notification of one’s own breast density were significant predictors of some knowledge about breast density.

Table 3 delineates the extent to which predictors associated with ‘some knowledge’ contributed to knowledge of specific factual information about breast density. Younger age, prior notification of breast density, and prior awareness of the term ‘breast density’ were significant predictors for knowing that breast density can mask cancer on a mammogram. With regard to knowledge about the potential requirement for further tests in women with high breast density, younger age, English as a language spoken at home, prior notification of breast density, and prior awareness of the term ‘breast density’ all demonstrated significance. However, only prior notification of breast density and prior awareness of the term ‘breast density’ significantly contributed to knowledge that high breast density is a risk factor for breast cancer.

Table 4 shows the degree to which factors predicted knowledge about common misconceptions related to breast density. English as a language spoken at home, prior breast density notification, and prior awareness of the term ‘breast density’ were predictive of participants’ knowledge that breast density is not related to how breasts look or feel. Similarly, the latter two predictors were associated with knowledge that breast density is not related to breast size.

Table 5 shows that eighty two percent of respondents expressed their interest in knowing their own breast density. Age, language spoken at home, and awareness of breast density were not predictors of whether women wanted to know their density.

## 4. Discussion

This study provides insight into awareness and knowledge about breast density among women in Australia. The findings reveal a significant deficit in fundamental knowledge of breast density, even though many of the participants were familiar with the term ‘breast density’. Moreover, women were largely unaware that breast density is a risk factor for breast cancer, despite many possessing knowledge about the masking effect. This study elucidates the factors that contribute to increased breast density knowledge, including younger age, English language spoken at home, prior breast density notification, and breast density awareness.

With ongoing discussion both in Australia and internationally as to the need for notification of breast density, this study highlights the informational needs of women to support accurate knowledge of breast density and the high degree of preference for knowing such information. Notably, South Australia is the second state in Australia, following Western Australia, to implement breast density notification as an integral component of the population-based screening program [3,20,21,25]. The Australian context differs from the United States, given that, by April 2015, a total of 22 states had enacted legislation mandating to notify women about breast density [27]. Likewise, information pertaining to breast density is distributed across the six jurisdictions in Canada in a comprehensive manner. Among them, five inform specifically women classified under category D directly, while one provides information to their respective physicians [28]. As notification in BreastScreen South Australia was commenced subsequent to the closing of this study, it is worth mentioning that the study cohort had not been generally exposed to density notification at the screening program before participating here. 

Regarding awareness among the study cohort, sixty percent of respondents had heard the term ‘breast density’ before. The level of awareness among South Australian women is notably lower compared to Western Australian women (>80%), where breast density notification has been more widespread [24]. 

The questionnaire revealed that approximately one-quarter of the study cohort had been notified of their own breast density, a finding that aligns with the results of a prior study conducted in the United States during a period when breast density notification was not mandated by legislation [29]. Less than 60% of participants correctly answered two or more questions out of five about breast density. Younger age and English language spoken at home were predictors of some knowledge, suggesting that targeting information to older women and those not fluent in English may provide opportunities to increase South Australian women’s knowledge about breast density. These findings underscore a fundamental knowledge deficit about breast density among Australian women. 

At the same time, awareness of breast density was a predictor of knowledge, suggesting that efforts in Australia to raise awareness have had an impact on knowledge. Similar findings were observed in a recent study conducted in Croatia [30]. This is in contrast with recent studies conducted in the United States. Variables including education, screening history, and preferred language were found to play a role in disparities in awareness but did not exert significant impact on knowledge levels [31]. Another US study reported that density notification may increase overall awareness; however, it may not have a discernible impact on increasing knowledge concerning the masking effect and the risk associated with breast cancer [32]. Further research is required to establish optimal approaches for raising awareness about breast density that leads to increased knowledge. 

In the current investigation, only 23% of women were aware that high breast density is a risk factor for breast cancer. This figure is nearly three times lower than that reported in a prior U.S. study, where breast density notification is mandated by law [33]. A European study suggests that awareness of breast cancer risk can have a favourable impact on breast screening rates [30]. Considering these findings, it is imperative to devise strategies for enhancing this important knowledge among women, aiming to promote greater participation in healthy lifestyles, breast awareness, and screening programs. 

Several states within the United States have already demonstrated positive outcomes by incorporating breast density notification into their population-based screening programs, leading to enhanced knowledge and awareness of breast density among women and increased participation in subsequent screening [33]. However, the exclusion of breast density information in breast cancer screening in Australia is viewed by consumers as failing to adequately address women’s ‘right to know’ and the enablement of their involvement in shared decision-making processes [3,34]. Interestingly, eighty-two percent of the respondents indicated a desire to ascertain their individual breast density, regardless of their level of awareness or whether they had been previously told their breast density by a health professional. This outcome reflects a broad interest in knowing one’s density, and is similar to the results observed in a pilot study conducted by BreastScreen South Australia [25]. 

Regrettably, there exists the risk of common misconceptions that may taint genuine knowledge. Common misconceptions regarding breast density, such as the belief that it can be diagnosed through touch or is associated with breast size, are prevalent. Notably, the current study identifies the key predictors dispelling these prevalent misconceptions among women. In this context, both prior breast density notification and breast density awareness play pivotal roles. While the current study suggests that awareness and notification is associated with increased knowledge about breast density, more communication is required to comprehensively dispel common misconceptions. Websites [35] and factsheets [36] that provide breast density information should be aware of these common misconceptions and actively seek to dispel them. For the most comprehensive information, personalised counselling within a dedicated radiologist-run breast density consultation may foster better understanding among women about breast density and augment their participation in shared decision-making [37]. 

However, radiologist consultation regarding breast density is not practical within population-based breast screening. The inclusion of general practitioners (GPs) and breast care nurses in this context could afford a more cost-effective and efficient support system for women with dense breasts. Given the inherent diversity in women’s knowledge levels and individual risk, we posit that personalised, one-on-one consultations is preferred, as a ‘one size fits all’ approach may not suffice. Given the limited understanding of mammographic density among Australian GPs [22], it is also imperative to explore approaches for enhancing their education and training. Well-structured and comprehensive training to GPs and breast care nurses could empower them with the requisite knowledge and competencies to guide women about critical information concerning breast density. This approach would empower women to be actively involved in making decisions on how best to manage their breast cancer risk and breast cancer screening in line with the National Women’s Health Strategy 2020–2030 of Australia [38] and Women’s Health Strategy for England 2022 [39].

This study addresses critical gaps within the extant literature, notwithstanding certain limitations. We enrolled women from a single centre and generalisability may be limited. Moreover, we did not collect data on the literacy status of women, despite its considerable influence on breast density awareness status [31]. This study cohort included only 6% of participants reporting speaking a language other than English at home. While there was a statistically significant difference in the odds of having some knowledge about breast density between the two language groups, the wide confidence interval may reflect the low proportion of participants speaking another language at home. Another constraint is our inability to elucidate the reasons for study non-participation among non-respondent women. It is plausible to speculate that non-participation might be influenced by factors such as coming from culturally and linguistically diverse (CALD) backgrounds and the need for interpreters for clinical appointments. The proportion of participation of women coming from CALD backgrounds was lower than the expected population from The Queen Elizabeth Hospital catchment area. A drawback of this kind of questionnaire for non-English speaking participants is the reliance on hospital interpreters, whose principal role is assisting patients in the context of their clinical consultation, not completing a research questionnaire. Interestingly, the majority of responses we received from CALD participants were when a family member proficient in English was present to assist, rather than a professional interpreter. To increase participation in women from CALD backgrounds, the study questionnaire should be translated into different languages.

The primary merit of this study is its capacity to discriminatively pinpoint influential predictors of common factual knowledge and misconceptions about breast density. Another notable strength of this study is its high response rate of 82%, which exceeds that of prior studies with similar [29] and even larger cohorts [24,33]. 

## 5. Conclusions

While knowledge of breast density in this Australian cohort is generally quite low, we have identified factors associated with increased knowledge. Women who had previously heard the term breast density had increased knowledge compared to those who had not, suggesting that current efforts to raise awareness are leading to better knowledge. Despite this, there are widespread misconceptions that must be actively dispelled, including the misunderstanding that breast density can be determined by touch. These findings support further efforts to raise awareness and promote education about breast density for women attending breast cancer screening. 

## Figures and Tables

**Table 1:** Characteristics of the study participants.

Variables	Frequency
Age (years) Mean (SD)	61.10 (±11.45)
Language spoken at home	
English	330 (94%)
Other	20 (6%)
Heard of breast density before	
Heard about breast density	215 (61%)
Never heard about breast density	135 (39%)
Accessible/Remoteness Index of Australia	
Highly accessible/Accessible/Moderately accessible	341 (97%)
Remote/Very remote	9 (3%)
Ever been told own breast density by any health professionals	
Yes	90 (26%)
No/Don’t know	260 (74%)
Breast density status	
High density (C/D)	234 (67%)
Low density (A/B)	116 (33%)
Want to know their own breast density	
Yes	286 (82%)
No/Don’t know	64 (18%)
Breast density knowledge status	
Low knowledge (Score 0–1)	151 (43%)
Some knowledge (Score 2 and above)	199 (57%)

**Table 2:** Multivariable binary logistic regression analysis for independent predictors associated with ‘some knowledge’ about breast density.

Independent Predictors	Comparison	OR (95% CI)	Global *p*-Value *
Age		0.97 (0.96, 0.99)	0.02 *
Language at home	English vs. Others	3.29 (1.23, 8.77)	0.02 *
Heard of breast density before	Yes vs. no/don’t know	4.05 (2.57, 6.39)	0.004 *
Ever been told about own breast density	Yes vs. no/don’t know	4.99 (2.76, 9.03)	0.004 *
SEIFA ^a^ decile		1.02 (0.94, 1.11)	0.95
ARIA ^b^	Accessible vs. Remote	1.06 (0.28, 4.00)	0.95
Number of previous mammograms ^c^		1.01 (0.82, 1.23)	0.95
Breast density status	High (C, D) vs. Low (A, B)	1.06 (0.67, 1.66)	0.95

Binary logistic regression modelling ‘some knowledge’ as response and ‘low knowledge’ as reference. a. SEIFA-Socio Economic Index, b. ARIA-Accessibility/Remoteness Index of Australia, c. in last 3 years. * Significant *p*-value after Benjamini–Hochberg adjustment. OR, odds ratio; CI, confidence interval.

**Table 3:** Multivariable binary logistic regression analysis for independent predictors of knowledge associated with common facts about breast density.

Independent Predictors	Do You Think Dense Breast Tissue Makes It More Difficult to See Cancer on a Mammogram?		Do You Think That, after a Mammogram, Sometimes Women May Require Further Tests because They Have Dense Breast Tissue?		Do You Think Having Breasts That Are Mostly Dense on a Mammogram Puts You at Increased Risk for Breast Cancer?	
	Frequency (%)	OR (95% CI) *p*-Value	Frequency (%)	OR (95% CI) *p*-Value	Frequency (%)	OR (95% CI) *p*-Value
Age		0.97 (0.95, 0.99) 0.001 *		0.96 (0.94, 0.98) <0.001 *		0.99 (0.97, 1.02) 0.55
Language at home						
English	158/330 (48%)	1.71 (0.66, 4.38) 0.27	202/330 (61%)	2.93 (1.14, 7.54) 0.03 *	77/330 (23%)	1.73 (0.49, 6.04) 0.52
Others	7/20 (35%)		7/20 (35%)		3/20 (15%)	
Ever been told about breast density by any health professionals						
Yes	73/90 (81%)	7.84 (4.37, 14.09) 0.001 *	72/90 (80%)	3.59 (2.03, 6.36) 0.001 *	35/90 (39%)	3.040 (1.79, 5.18) 0.004 *
No/Don’t know	92/260 (35%)		137/260 (53%)		45/260 (17%)	
Heard of breast density before						
Yes	131/215 (61%)	4.64 (2.88, 7.45) 0.001 *	148/215 (69%)	2.68 (1.72, 4.18) 0.001 *	60/215 (28%)	2.23 (1.27, 3.90) 0.02 *
No/Don’t know	34/135 (25%)		61/135 (45%)		20/115 (17%)	

Binary logistic regression modelling correct answer as response and wrong answer as reference. * Significant *p*-value after Benjamini-Hochberg adjustment. OR, Odds Ratio; CI, Confidence interval.

**Table 4:** Multivariable binary logistic regression analysis for independent predictors of knowledge associated with common misconceptions about breast density.

Independent Predictors	Do You Think Breast Density Can Be Determined by Feel or Touch?		Do You Think Women with Large Breasts Are More Likely to Have Dense Breast Tissue Than Women with Small Breasts?	
	Frequency (%)	OR (95% CI) *p*-Value	Frequency (%)	OR (95% CI) *p*-Value
Age		0.99 (0.97, 1.01) 0.28		0.98 (0.97, 1.02) 0.69
Language at home				
English	101/330 (31%)	3.97 (0.90, 17.43) 0.09	91/330 (28%)	2.37 (0.83, ∞) 0.004 *
Others	2/20 (10%)		0/20 (0%)	
Ever been told own breast density by any health professionals				
Yes	36/90 (40%)	1.92 (1.16, 3.18) 0.02 *	33/90 (37%)	2.11 (1.20, 3.39) 0.01 *
No/Don’t know	67/267 (25%)		58/260 (22%)	
Heard of breast density before				
Yes	78/215 (36%)	2.51 (1.50, 4.20) 0.004 *	68/215 (32%)	2.25 (1.32, 3.84) 0.004 *
No/Don’t know.	25/135 (19%)		23/135 (17%)	

Binary logistic regression modelling correct answer as response and wrong answer as reference. * Significant *p*-value after Benjamini–Hochberg adjustment OR, odds ratio; CI, confidence interval.

**Table 5:** Multivariable binary logistic regression analysis for independent predictors of interest to know own breast density.

Variables	You Are Waiting to Have a Mammogram at TQEH, and This Will Show Your Breast Density. Would You like to Be Told Your Breast Density?	
	Frequency (%)	OR (95% CI) *p*-Value
Age		0.99 (0.96, 1.01) 0.28
Language at home		
English	271/330 (82%)	1.53 (0.54, 4.38) 0.43
Others	15/20 (75%)	
Ever been told about breast density by any health professionals		
Yes	80/90 (89%)	2.09 (1.02, 4.32) 0.14
No/Don’t know	206/260 (79%)	
Heard of breast density before		
Yes	182/215 (85%)	1.64 (0.95, 2.84) 0.14
No/Don’t know	104/135 (77%)	

Binary logistic regression modelling ‘want to know’ as response and do not want to know as reference. *p*-value after Benjamini–Hochberg adjustment. OR, odds ratio; CI, confidence interval.

## Data Availability

Data will be made available upon request.

## Supplementary Materials

The following supporting information can be downloaded at: https://www.mdpi.com/article/10.3390/cancers16050893/s1, study questionnaire.
