# Supplementary material for: Factors Associated with Increased Knowledge about Breast Density in South Australian Women Undergoing Breast Cancer Screening
Source: Cancers (Basel). 2024 Feb 23;16(5):893. doi: 10.3390/cancers16050893 (PMC10930536; doi:10.3390/cancers16050893)
Supplement: Supplementary file 1 [file cancers-16-00893-s001.zip › cancers-2865541-supplementary.pdf]

Government of South Australia
SA Health

THE UNIVERSITY of ADELAIDE

QUESTIONNAIRE

Breast/Endocrine Surgical Unit - The Queen Elizabeth Hospital

**Title:**	Assessing Patients' Knowledge about Breast Density
**Protocol Number:**	15681
**Principal Investigator(s):**	Associate Professor Wendy Ingman, Dr Avisak Bhattacharjee, Dr Pallave Dasari, Dr David Walsh, and Ms Leigh Hodson

---

- What language do you speak at home?

  English Other

Please specify ____________________

- Are you being assisted by an interpreter today?

  Yes No Don't know

Breast density is a measure of how white the breasts appear on a mammogram. Breasts that appear mostly dark on a mammogram are low density, breasts that appear mostly white on a mammogram are high density.

- Before now, had you ever heard of breast density?

  Yes No Don't know

- Do you think women with large breasts are more likely to have dense breast tissue than women with small breasts?

  No, strongly disagree No, disagree Unsure Yes, agree Yes, strongly agree

- Do you think breast density can be determined by feel or touch?

  No, strongly disagree No, disagree Unsure Yes, agree Yes, strongly agree

- Do you think dense breast tissue makes it more difficult to see cancer on a mammogram?

  No, strongly disagree No, disagree Unsure Yes, agree Yes, strongly agree

Please complete both sides of the page.

- Do you think that, after a mammogram, sometimes women may require further tests because they have dense breast tissue?

  No, strongly disagree   No, disagree   Unsure   Yes, agree   Yes, strongly agree

- Do you think having breasts that are mostly dense on a mammogram puts you at increased risk for breast cancer?

  No, strongly disagree   No, disagree   Unsure   Yes, agree   Yes, strongly agree

- Have you ever been told by a health professional that your breast tissue is dense?

  Yes   No   Don't know

- If yes, who told you? A GP, BreastScreen, Radiologist, other, please specify____________________

- Have you ever had a mammogram before?

  Yes   No   Don't know

- You are waiting to have a mammogram at TQEH, and this will show your breast density. Would you like to be told your breast density?

  Yes   No

- The Queen Elizabeth Hospital is conducting a number of studies about breast density. If you are eligible for any studies, would you like a researcher to contact you?

  Yes   No
